# Building Clinical Trials Capacity for Tuberculosis Drugs in High-Burden Countries

**DOI:** 10.1371/journal.pmed.0040302

**Published:** 2007-11-06

**Authors:** Neil Schluger, Unni Karunakara, Christian Lienhardt, Thomas Nyirenda, Richard Chaisson

## Abstract

The long duration of TB therapy, the high prevalence of HIV coinfection, and the growing prevalence of drug-resistant TB underscore the urgent need for more effective treatments.

## Current Treatment for Tuberculosis and Research Agenda

The standard preferred regimen for the treatment of drug-susceptible tuberculosis (TB) around the world consists of a two-month induction phase of isoniazid, rifampin, pyrazinamide, and ethambutol followed by a continuation phase of four months of isoniazid and rifampin. This regimen has several advantages: (1) it is essentially completely effective in achieving sputum culture conversion; (2) it is relatively inexpensive and universally available; (3) the relapse rate is low, at 3%–5%; and (4) it can be administered largely intermittently. But the regimen has significant limitations, listed in Box 1.

These limitations can only be overcome by building TB clinical trials capacity. Trials are urgently needed to find regimens that are shorter, less toxic, and effective against multidrug-resistant TB (MDR-TB; Figure 1) and extensively drug-resistant TB (XDR-TB). TB treatment regimens must be tested in special populations: people with HIV infection, including those on antiretroviral (ARV) therapy, children, and patients with extrapulmonary TB. Because of the rapidly evolving nature of the global tuberculosis epidemic, with increasing numbers of HIV-associated cases of TB, more drug resistance, and several new zdrug candidates, we will need to find ways to evaluate new regimens more quickly, for example through identifying new, robust biomarkers.

**Figure 1:**
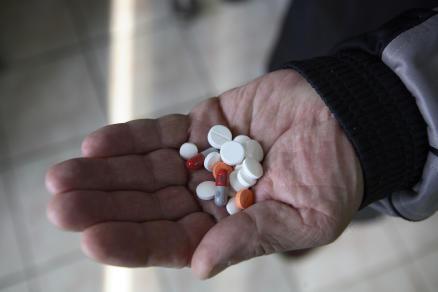
A Patient with MDR-TB Holds the Dose of Drugs He Must Take Every Day This patient checked into the hospital in November 2006 and in January 2007 he tested negative; if his progress continues, he could be sent home to finish his treatment. (Photo: Jean-Marc Giboux)

**Summary Points**

- The long duration of TB therapy, the high prevalence of HIV coinfection, and the growing prevalence of drug-resistant TB strains underscore the urgent need for better and more effective treatment approaches.
- To improve TB treatment in the near future, a large number of clinical trials should be carried out to examine alternative regimens using existing drugs (dosage changes, frequency of dosing, etc.) and the modest but growing pipeline of new compounds.
- The capacity to conduct a serious clinical trials agenda in high-burden countries needs to be built up as soon as possible.
- Funding on the order of US$300–US$500 million annually will be needed to conduct such a clinical trials agenda, including the evaluation of new drugs.
- Direct investment must be made in the infrastructure needed to conduct trials, rather than taking a product-byproduct approach.

A few recent, small-scale trials provide an indication of possible new treatment regimens, but many types of investigations still need to be carried out (Table 1). The promising early findings and unresolved (or even unasked) questions require further examination and refinement, which could be done today if the resources were available to build clinical trials capacity in high-burden countries.

**Table 1:**
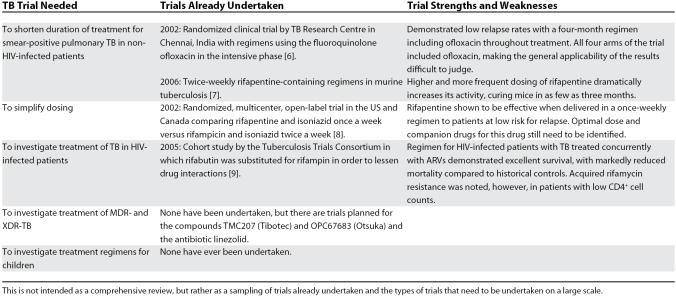
Tuberculosis Trials Needed and Undertaken

## What Is Needed to Build Clinical Trial Capacity

The experience of the British Medical Research Council's TB research units provides a useful template for planning the renewal of capacity for clinical trials in TB [1]. Over 40 years, the Medical Research Council evaluated all the drugs currently in use for the treatment of TB. To do this, they tested roughly 250 different regimens, enrolling over 25,000 patients in trials. Why so many? Patients with TB are treated with regimens, not individual drugs. Even if a single new drug is approved for the treatment of TB, finding its optimal dosage, dosing frequency, and companion drugs will require several trials.

Many questions need to be answered through a variety of clinical trials as described in Table 2. For example, moxifloxacin and gatifloxacin are promising compounds that provide faster time to culture conversion in patients with pulmonary TB [2]. Moxifloxacin appears in animal models to be more effective when substituted for, rather than added to, isoniazid in the intensive phase of chemotherapy. Also, in a phase II study conducted in 217 patients with TB in South Africa, substitution of ethambutol with gatifloxacin or moxifloxacin was associated with enhanced early and late sterilizing activity [3]. The optimal rifamycin with which to combine therapy may be rifapentine rather than rifampin [4].

**Table 2:**
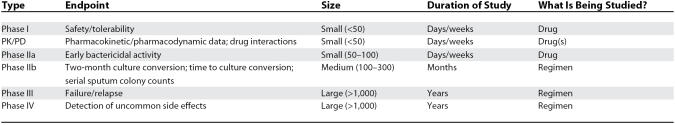
Types of Tuberculosis Clinical Trials

Sample sizes for TB trials (especially phase III trials) are quite large because current TB treatment, though complex and prolonged, is associated with a low relapse rate. So significant numbers of patients are required to ensure with a high degree of confidence that newer regimens are at least as effective and associated with the same or lower rate of adverse drug effects as existing regimens. Based on the analysis of previous trials conducted by the British Medical Research Council and the TB Trials Consortium of the United States Centers for Disease Control and Prevention, capacity must exist to enroll 3,000–5,000 patients per year in phase II and III trials, for each of the seven to ten compounds currently in clinical development, if TB treatment is to be substantially improved in the next five to ten years. However, neither the infrastructure nor financial support exists on the scale needed to carry out these important high-quality clinical trials.

### Infrastructure needs

TB trials can be effectively carried out by collaborations between front-line providers in high-burden countries and academic institutions that can provide technical support and assistance in study design, data analysis, drug procurement, and training of research personnel. The regulatory and ethical requirements that now exist for clinical research demand that trials be carried out according to high standards, without compromise on scientific or moral grounds.

Box 2 lists the factors that are needed to properly conduct TB trials. These needs are especially acute in many high-burden countries where there is no established research infrastructure and where capacity for ethical and regulatory review may be limited and may face special challenges. Effective programs for delivering TB treatment exist in many places, but such programs are not equivalent to a clinical trials infrastructure. Furthermore, creating an enduring infrastructure would increase the speed and efficiency with which trials can be conducted. Several consortia are currently managing to carry out clinical trials, but with minimal coordination and inadequate financial support. With greater support and coordination, these consortia can expand trials capacity through standardized approaches to trial design, by providing training (especially in high-burden countries), and by establishing working relations with the regulatory agencies overseeing the introduction of new drugs in locations around the world.

Limited capacity exists today for conducting clinical trials at good clinical practice standards. The Global Alliance for TB Drug Development recently completed a survey of 51 potential sites where clinical trials for TB might be carried out. Preliminary results indicate that only a few sites around the world, most notably Rio de Janeiro, Brazil and Durban, South Africa, have the needed components and experience to begin enrolling patients in significant numbers.

### Funding

Funding for overall TB drug development is at pitifully low levels, and for clinical trials it is even worse. A report by the Treatment Action Group describes a comprehensive global survey of funding for TB research [5]. Around the world in 2005, only US$120 million was spent for research on TB drug development, and the amount of that total which was devoted to actual clinical trials was no more than US$20–US$30 million. The US National Institutes of Health (NIH) support the Tuberculosis Research Unit, whose primary mission is to study immune mechanisms in patients with TB rather than carrying out clinical trials of TB drugs. The US Centers for Disease Control and Prevention created and fully supports the TB Trials Consortium with a budget of only US$9.2 million a year. The European and Developing Countries Clinical Trials Partnership has spent very little on TB up until now, but expects to spend an extra sum of US$38 million by 2010, with additional funds to support clinical trials capacity development in African trials. Monies from some pharmaceutical companies, and from the Global Alliance for TB Drug Development (which is mostly funded through the Bill & Melinda Gates Foundation with additional support from a few European countries), comprise the bulk of the rest of the funding support.

**Box 1. Limitations of the Standard TB Treatment Regimen**

- Adverse effects are common—the most important adverse effect is hepatotoxicity, which can be serious and fatal
- Difficult to administer concurrently with many ARV drugs
- High rates of nonadherence if self-administered
- Relapse rates in certain subsets of patients (e.g., those with extensive cavitary infiltrates) may be 15% or more
- The regimen is not useful against MDR or XDR strains.

The Treatment Action Group report points out that in the United States, the NIH alone spent US$2.9 billion on research related to HIV/AIDS in 2005. At least US$300 million of this funding directly supported clinical trials, mainly through large and well-organized consortia such as the AIDS Clinical Trials Group and the Community Programs for Clinical Research on AIDS. Biodefense spending by NIH exceeds the total expenditure on research for TB, which is the only disease on their list of perceived threats that actually takes millions of lives each year. In 2005, for example, NIH spent US$187 million and US$183 million on research on smallpox and anthrax, respectively, and only US$157 million on TB.

Based on the experiences of groups currently conducting trials, the estimated costs for the TB trials work needed to create new regimens and regimens useful against MDR- and XDR-TB considerably exceed current spending. Based on the actual costs of trials conducted in the past few years by the TB Trials Consortium, the European Union–funded consortium of ten European and African institutions (the OFLOTUB consortium), and the Johns Hopkins TB Research Center, establishing infrastructure for 25 sites with appropriate clinical, laboratory, and regulatory expertise will be on the order of US$1–US$2 million per site per year, while costs for actual trials will range between US$4,000–US$12,000 per patient, depending on the exact location of the study (personal communication, N. Schluger, C. Leinhardt, and R. Chaisson).

## Conclusion

An urgent and massive expansion of clinical trials capacity is needed to carry out vital research to accelerate the development and evaluation of new TB drugs, including those active against MDR-TB. To conduct the clinical trials agenda described above, including the evaluation of new drugs, funding of at least US$300–US$500 million annually is needed. It is also important to make direct investment in the infrastructure needed to conduct trials, rather than taking a product-by-product approach. Current global plans for TB drug development assume that specific funding is tied to individual drugs in the pipeline and does not take into account the need for trials for MDR-TB. We believe that this product-by-product approach will lead to inefficiency, redundancy, and wasted time, and will fail to address the important question of how to construct regimens. Independent support for clinical trial sites that are prepared to undertake a variety of studies is essential to ensure that new regimens are developed as quickly as possible. A strategy where strong clinical trial sites are funded to build capacity to support weaker centers will ensure increased and sustained capacity to conduct more trials in less time.

**Box 2. Infrastructure Needed in High- and Low-Burden Countries for Conducting TB Trials**

Building research capacity and infrastructure in both high- and low-burden countries requires a significant initial investment of funds, but is extremely cost-effective in the long run, as it allows for the creation of a stable and well-trained cohort of investigators and nurses as well as mechanisms for rapid development and evaluation of a series of research protocols, without the need to recreate these components each time a trial is proposed.

**Infrastructure Needed in Both High- and Low-Burden Countries**

- Physicians and nurses experienced in caring for patients with TB and trained in clinical research
- Field workers to assist with therapy and assure that study regimens are followed correctly
- Institutional review boards/ethics committees that review proposals in an informed and timely manner
- Regulatory experts to aid in moving new compounds through the varied and complex regulations governing the use of experimental drugs in many different countries
- Well-equipped and functioning laboratories with capacity for performing culture and susceptibility testing on clinical isolates (or capacity for shipping to central laboratories that have this capacity)
- Site monitoring capability and the logistical capacity to ensure that the quality of all of the above is maintained throughout

**Infrastructure Needed in Low-Burden Countries Only**

- Experienced biostatisticians to support trial design, statistical analysis, and data monitoring

A large number of clinical trials with the potential to significantly improve TB treatment in the very near future can be carried out right now. As new drugs move through the pipeline, the trials agenda will grow rapidly. A network of existing consortia should be created to develop uniform standards and platforms for conducting this trials agenda and to coordinate the various trial activities. Most important, a massive increase in funding, from public and philanthropic sources, is urgently needed for clinical trial sites, in order to reduce the global burden of TB and to improve the lives of patients with TB.

## Abbreviations

ARV: antiretroviral
MDR-TB: multidrug-resistant tuberculosis
NIH: National Institutes of Health
TB: tuberculosis
XDR-TB: extensively drug-resistant tuberculosis
